# Syntactic complexity in translated and non-translated texts: A corpus-based study of simplification

**DOI:** 10.1371/journal.pone.0253454

**Published:** 2021-06-24

**Authors:** Kanglong Liu, Muhammad Afzaal

**Affiliations:** 1 Department of Chinese and Bilingual Studies, The Hong Kong Polytechnic University, Hong Kong, China; 2 Institute of Corpus Studies and Applications, Shanghai International Studies University, Shanghai, China; University of Sao Paulo, BRAZIL

## Abstract

This study approaches the investigation of the simplification hypotheses in corpus-based translation studies from a syntactic complexity perspective. The research is based on two comparable corpora, the English monolingual part of COCE (Corpus of Chinese-English) and the native English corpus of FLOB (Freiburg-LOB Corpus of British English). Using the 13 syntactic complexity measures falling into five subconstructs (i.e. length of production unit, amount of subordination, amount of coordination, phrasal complexity and overall sentence complexity), our results show that translation as a whole is less complex compared to non-translation, reflected most prominently in the amount of subordination and overall sentence complexity. Further pairwise comparison of the four subgenres of the corpora shows mixed results. Specifically, the translated news is homogenous to native news as evidenced by the complexity measures; the translated genres of general prose and academic writing are less complex compared to their native counterparts while translated fiction is more complex than non-translated fiction. It was found that mean sentence length always produced a significant effect on syntactic complexity, with higher syntactic complexity for longer sentence lengths in both corpora. ANOVA test shows a highly significant main effect of translation status, with higher syntactic complexity in the non-translated texts (FLOB) than the translated texts (COCE), which provides support for the simplification hypothesis in translation. It is also found that, apart from translation status, genre is an important variable in affecting the complexity level of translated texts. Our study offers new insights into the investigation of simplification hypothesis from the perspective of translation from English into Chinese.

## Introduction

The quest for translation universals (TUs) using corpus-based translation methods spearheaded by Baker [1, 2] paved the way for a new wave of academic research into the unique features of translational language. The many developments undertaken by Baker in translation studies included a change in orientation from the source text to target system and a shift from exploring equivalence to describing translation norms [3]. Hence, thanks to the widespread use of corpora in the field of translation studies, the trends in translation moved from investigations on individual and idiosyncratic features to a scientific study of a general translation phenomenon.

Baker [1] defined TUs as “the universal features which typically occur in translated text rather than original utterances and which are not the result of interference from specific linguistic systems”. Since Baker’s formulation of the TUs hypothesis, the concept of TUs has engaged translation researchers and scholars on a debate of whether translation could have universals [4–6]. Traditionally, divergences between translated and non-translated texts in target language (TL) have led to translation being portrayed stereotypically and derogatively as “translationese” which is caused by the translator’s incompetence [7]. Translation has long been regarded as a deviant, derivative, and unnatural language variety that is not comparable to native writing, and the focus on equivalence or correspondence in prescriptive translation studies has also led translators to be perceived as ‘invisible’ [8]. For a long time, translation has been accorded a low and marginal status in language studies which prioritizes creative writing over translation under the traditional convention of emphasizing the author’s instead of translator’s style [9]. Due to its marginal status, the notion of TUs has shared similarities with a number of concepts, such as “inter-language” [10], “third language” [11], “third code” [12] and also “hybrid language” [13]. Nonetheless, the quest for TUs has a long history and has contributed to the establishment of translation studies as an independent discipline. This line of research has helped provide insights into identifying what exactly translating is, and unveil the unique features of translational languages. Despite the controversies surrounding the concept, corpus-based research into TUs has been one of the most important methodological advances in translation studies [14].

Although corpus-based TUs research is still controversial as compared to other well-grounded and schematized corpus-based translation studies, TUs has been extensively and fruitfully investigated in the field of translation studies. In the field of translation studies, the concept of TUs has been questioned, scrutinized or even rejected by researchers [15, 16]. For example, some researchers have been critical of the concept of TUs for the unscientific use of the concept [5, 6] and some have been cautious of the existence of universal features inherent in every translation [17–19], while others have called for the creation of probabilistic translation laws instead of universals to promote translation research [20]. Taking the view that TUs should only be conceived as being universals if they instantiate in translations alone without occurring in other forms of texts, Pym [21] argues that the term “universal” should be reserved for actual, concrete linguistic phenomena measurable by scientific research tools. However, Chesterman [22] contended that the significant issue with TUs is terminological which should be understood in a weaker sense like general tendencies or patterns or generalizations in translation research. Despite views to the contrary, the growing interests in translation research has continued to captivate the interests of researchers working in corpus-based translation studies. These productive efforts to uncover the unique features of translational language include “simplification (translation tends to simplify language use in comparison to native texts) [23, 24], “explicitation (translation tends to state the information in a more explicit form than the native texts” [25, 26], “normalization or conservation (translation tends to conform to linguistic characteristics typical of the target language” [27, 28], and “levelling out (translation tends to be more homogeneous than native texts)” [29]. Although there has been extensive research on TUs using corpus-based translation studies (CBTS), the fundamental issues on TUs remain uncharted. Questions pertaining to how factors such as social, pragmatic, and cognitive mechanisms shape translation remain to be addressed [3], as do exploration of ways in how these parameters affect the process of translation. Viewing translation as a social translation, Pym [6] explained that many translation features are connected to translator’s risk-aversion as translation is in nature a mediation activity involving certain socio-communicative risks. On the cognitive side, there are also two models concerning the explanation of these translation features: the relevance-theoretical model [30, 31] that is based on Relevance Theory [32] and the cognitive grammatical model, also known as the gravitational pull hypothesis, [33, 34] that draws on bilingualism and Second Language Acquisition.

The development and controversies of the concept of TUs in translation research are reviewed here because simplification research has often been pursued under the umbrella term of TUs. Although theoretical and conceptual variations exist as to the scientific use of term, the TUs concept has motivated a number of corpus-based studies and greatly advanced translation research. The goal of the current study is not aimed at simply confirming or refuting the existence of simplification as a TUs candidate, instead, TUs is used as a starting point to probe into the complex nature of translational language especially when genre is brought into the picture. The paper is structured as follows. Section 1 is a general introduction providing the necessary background for the current study. Section 2 provides a review of relevant corpus-based studies on simplification, highlights some gaps in this line of research and presents the research questions. Section 3 examines the use of syntactic complexity in language and translation research. The design of the COCE corpus and the methodology of the study are described in Section 4. Section 5 reports the results and Section 6 attempts to interpret the results in relation to some previous studies on translational simplification. Lastly, Section 7 concludes by discussing the implications and caveats of our approach and outlining directions for future research.

## Previous studies on simplification

In the field of translation studies, TUs has been predominantly studied from a Eurocentric perspective [4, 19, 23]. The research on TUs has largely been confined to closely-related European languages, and the linguistic features may not be as distinctively dissimilar as in genetically distant languages such as English and Chinese [24]. Previous studies pertaining to TUs have identified comparative overuse/underuse and different uses in lexical, syntactic and stylistic properties in translated and non-translated texts. These studies have provided some support that translated language is distinct from non-translated language with respect to a number of linguistic and syntactic features.

However, the most debated and widely discussed translational universal is perhaps simplification, which has attracted the attention of a number of scholars [24, 35]. Simplification is defined as “the idea that translators subconsciously simplify the language or message or both” [36]. The existing literature on simplification in the field of translation studies has focused primarily on identifying the linguistic features attributable to translation-specific influences. Along the years, various linguistic features have been used for studying simplification. For example, lexical simplification has been described as “making do with fewer words” [37], using informal, colloquial and modern lexis to translate formal, literate and archaic words in the source text [38] as well as a lower type-token ratio in the translated texts [39]. While examining the notion of TUs, Laviosa [35] identifies some evidence in support of the simplification hypothesis in lexical patterns in English translation. Specifically, the features of translated texts include: (1) a relatively lower percentage of content words compared to grammatical words; (2) a higher proportion of high-frequency words to low-frequency words; (3) the list head of a corpus of translated texts accounts for a larger area of the corpus; and (4) the list head of a corpus of translated texts contains fewer lemmas. On the contrary, Mauranen [40] points out that translation also contains some “strange strings”, or odd collocations, which contradicts the simplification hypothesis. Despite the amount of research on the subject, simplification remains controversial in comparison to other TUs candidates. The findings tend to be ambivalent and the existing literature has reported contradictory results, e.g. greater mean sentence length [35], untypical collocations [41] and more frequent use of modifiers [42].

The research on simplification has some methodological issues. First, this line of research predominantly focuses on lexical simplification while ignoring simplification at the syntactic or stylistic levels. Second, the measures chosen to study simplification are often randomly selected and more often than not to confirm the simplification hypothesis. Third, statistical methods have rarely been used to test whether the observed differences are statistically significant. In recent years, the use of the multivariate statistical methods in order to understand the phenomenon of TUs has received more attention [43, 44]. The methodological weaknesses of TUs research have been a major limitation of many previous studies which rarely included an analytical model to provide a scientific account of simplification in translated texts. Moreover, like other corpus-based TUs studies, representativeness of the corpus remains a major weakness hampering this research field. For example, the TEC Corpus [35], which comprises four text types of biography, news, fiction and magazine, is unbalanced and skewed disproportionately towards the literary genres, thus lacking representativeness. The compilation of a representative balanced corpus is an important step for systematically investigating the simplification phenomenon in translation. Researchers have also pointed out that one major weakness of TUs research is that the assumed features including simplification are considered independent of genre or language pair which can play key roles in shaping the makeup of translated language [18, 45]. Genre as a variable in translated language was rarely investigated in the quest of TUs which mainly focuses on the factor of “translation status”. For this reason, little progress has been made regarding the relationship between genre and the features of translation. This is also the case with the investigation of the simplification features. To some extent, the relationship between genre and the features of translational language has not received much attention [45]. Based on a comparable corpus of translated and original English produced in South Africa, Kruger and Van Rooy [45] also found that simplification tends to occur in translational language of more informal and creative genres, but not so much a prevalent feature in more informational genres. This reflects that genre can be a potential variable in shaping the profiling of translation language and should be taken into account in investigating simplification. This is one of the reasons that the current study adopts a balanced corpus design by taking genre variation into consideration.

Few studies have approached simplification by studying the syntactic complexity of translated versus non-translated texts. Although the term simplification has been frequently used and discussed in the field of translation studies, translated vs non-translated texts have seldom been evaluated using syntactic complexity measures. In fact, under the TUs framework, syntactic complexity is worthy of serious study because a methodical account of the syntactic features of translational language is vital to understand the important mechanism and values of the process of translation. A systematic study on syntactic complexity can provide significant insights into the simplification issue, and probe into the syntactic features of translation as opposed to non-translation.

The purpose of the present study is to apply the complexity measures to study simplification in translated texts. We contend that syntactic complexity is an important construct for probing into simplification and knowing the real nature of the translation products. In order to investigate whether and to what extent translated English texts tend to be simpler than non-translated texts (Chinese into English translation), the study aims to addresses the following research questions:

1. How are translated texts and non-translated texts different in terms of syntactic complexity?
2. Does syntactic complexity differ between translated texts and non-translated texts within the same genre?
3. Based on the above analysis, to what extent can the simplification universal be confirmed in translated texts?

## Syntactic complexity in language and translation research

Syntactic complexity is manifest in second language writing in terms of how varied and sophisticated the production units or grammatical structures are, which has been considered an important construct in second language teaching and research [46]. Syntactic complexity is viewed as an important construct of a language user’s competence in the target language. As an important construct of language learning and acquisition, syntactic complexity which concerns the variety and sophistication of linguistic units or grammatical structures has been fruitfully investigated in language learning research [47, 48]. Specifically, researchers would adopt corpus-based cross-sectional studies to compare the differences in syntactic complexity in length of production unit, amount of subordination, amount of coordination and degree of phrasal complexity between native and nonnative writing [47, 49, 50]. In a number of studies, it was found that nonnative writing is significantly different from native writing with an overrepresentation of coordination and complex phrases and underrepresentation of subordination [51]. In addition, it was found that nonnative writing contains more shorter clauses, sentences and T-units and fewer nominal phrases than native writing [47].

Due to the disparate development of second language acquisition and translation research, the field of translation studies has largely ignored the developments in second language acquisition. Such a situation may be related to the European tradition which attaches more importance to “direct translation” (translators work from a foreign language into their mother tongue) rather than “inverse translation” (translators work from their mother tongue into a foreign language). The opinion that translating from one’s own language does not have any but pedagogical purpose is deeply rooted in Europe, as evidenced by the widespread practice by international organizations which accept only the translation into the mother tongue [52]. On the other hand, China has a long history of inverse translation, and today translation out of Chinese is even more commonly practiced than ever [53]. Due to the different traditions, many English translations are done by Chinese translators from their mother tongue (L1) into a foreign language (L2), which is also the case with COCE. Though most of the translators are competent bilinguals, research has shown that translation directionality still serves as an essential factor in affecting the makeup of translated texts [54]. It should be noted that corpus-based translation research in the quest of TUs in European settings has largely ignored the variable of translation directionality. In a recent research utilizing a multilingual corpus featuring plenary speeches at the European Parliament with English translated texts together Italian and French source texts, it was found that source language serves a key variable in affecting the simplification level of translated texts [55]. The current research which examined translated English with Chinese as the source language (a typologically different language than English) will yield some interesting findings than most studies which are based on European languages.

While there is considerable research devoted to the investigation of syntactic complexity in ESL and EFL settings, relatively little attention has been paid to the use of such measures by translation researchers despite their connection to simplification research. As far as simplification is concerned, translation researchers more often studied lexical simplification by using a number of isolated features (see Section 2). In view of the shared nature between translated language and second language output and the merits of syntactic complexity measures, corpus-based research on simplification would be remiss not to make use of such measures that have been proved effective in ESL and EFL context. A systematic study into the specific complexity patterns of translated language would yield more findings than previous research using traditional parameters.

Therefore, in order to fill the existing gaps in simplification research, the current study adopts an interdisciplinary approach enlightened by similar research in ESL and EFL to probe into the translational simplification from the perspective of syntactic complexity. It is envisaged that the methods for measuring syntactic complexity in EFL can be effectively applied to translation studies to yield a scientific and systematic description of the unique features of translation. In order to get a complete understanding of syntactic complexity in translated and non-translated texts, the present study adopts the syntactic complexity measures generated by the L2 syntactic complexity analyzer (henceforth L2SCA) [46]. The measures contain five major components including length of production unit, amount of subordination, amount of coordination, phrasal complexity and overall sentence complexity. This is in line with the recent development that syntactic complexity is increasingly viewed as a “multidimensional construct” comprising of a number of global (e.g., mean length of sentence), clausal (e.g., subordinated and coordinated phrases per T-unit) and phrasal (e.g., complex nominal per T-unit) subconstructs [50, 56]. The five subconstructs were measured in the current study to examine whether translated texts are syntactically simpler than non-translated texts from a comprehensive perspective.

## Corpus analysis

As has been mentioned in the foregoing review, there remains gaps in corpus-based translation studies on simplification. With the aim of investigating the simplification features in translated texts, the current study was based on two corpora, namely, Corpus of Chinese into English (COCE) which was designed as a counterpart to the already existing corpus of Freiburg-LOB Corpus of British English (FLOB) [57]. The compilation of COCE is supported by the joint ESRC (UK)–RGC (Hong Kong) research project “Comparable and Parallel Corpus Approaches to the Third Code: English and Chinese Perspectives” (ES/K010107/1). This project is led by Dr Richard Xiao and Dr Andrew Hardie at CASS in collaboration with Dr Dechao Li and Professor Chu-Ren Huang of the Hong Kong Polytechnic University. As corpus contains a large amount of naturally occurring language data, it has become an ideal data source for investigating language and language use [58]. Likewise, corpus-based investigation of simplification has proved more promising than the traditional textual methods in view of its capability of handling a large amount of data. The current study attempts to systematically investigate the syntactic complexity between translated (COCE) and non-translated native English (FLOB) with the aim of exploring the simplification features in translated texts.

COCE is a parallel balanced corpus that matches closely in size and composition as FLOB. The corpus contains two parts, i.e., Chinese source texts and the correspondent English translations. The current research is based on the English monolingual part of the corpus. Hu, Xiao and Hardie [43] used COTE (Corpus of Translated English) which is a translational English corpus whose sources texts come from a number of different languages. In comparison to COTE, COCE is different and unique in two different ways. Firstly, COCE is a parallel corpus with Chinese source texts and English translation aligned at the sentence level. One significant advantage of such a design is that it ensures the corpus contains real translations (source texts vs target texts) without being polluted by some other substandard texts such as abridged translation, adapted translation, or pseudo translation. Second, as a parallel Chinese-English corpus, the representativeness is greatly enhanced and the findings can be explained in relation to the specific context of Chinese-English translation.

COCE contains 500 text samples of around 2000 words covering 4 major genres and 15 subgenres. (The total token count is somewhat higher than one million because the punctuation marks are also counted as separate tokens; however, we did not count punctuation marks as words when measuring the 2,000-word sample length, following the usual practice of the Brown Family.) Table 1 shows the detailed description of the English component of COCE, including the specific genres with numbers of texts, tokens and types, TTR and STTR. Following Baker’s comparable corpus approach [1], this study compares the translational English component of COCE with the comparable original English corpus (FLOB) in order to 1) systematically investigate the extent to which translational English differs from native English in syntactic complexity; 2) examine translational simplification.

**Table 1:** Design scheme of COCE and statistical facts of its English component.

Register	Code	Genre	Texts	Tokens	Types	TTR and STTR*
News	ABC	News (reportage, editorial, review)	88	183101	15617	11.72(47.57)
General Prose	D	Religious writing	17	34968	5610	6.23(39.90)
	E	Skills, trades and hobbies	36	78702	7608	10.34(35.99)
	F	Popular lore	48	91134	9342	9.76(42.73)
	G	Essays and biography	75	161455	15380	10.50(44.86)
	H	Miscellaneous	30	61616	5806	10.61(33.99)
Academic writing	J	Academic prose	80	164704	14939	11.03(41.07)
Fiction	K	General fiction	29	59815	7719	7.75(44.33)
	L	Mystery and detective stories	24	49566	6369	7.78(45.26)
	M	Science fiction	6	12380	3235	3.83(47.58)
	N	Adventure fiction	29	58869	7063	8.33(43.41)
	P	Romantic fiction	29	59834	7278	8.22(43.80)
	R	Humor	9	18825	3818	4.93(45.10)
			500	1034969	40874	25.32(42.93)

* TTR stands for type/token ratio, which is calculated by dividing the total number of distinct words (types) by the total number of items (tokens). STTR stands for standardized TTR which is calculated by dividing the tokens and the types after every thousand words in each text file.

### Corpus design

As a parallel corpus designed to be comparable to FLOB in genre and size, COCE comprises news (A-C, 17.6%); (2) general prose (D-H, 41.2%); (3) academic writing (J, 16.0%); and (4) fiction (K-R, 25.2%). Syntactic complexity is measured between translational and non-translational English as a whole and across all the four genres. The variation of genres in such a corpus design can provide a systematic analysis into the syntactic complexity of translational language, thus enabling us to get a better understanding of the simplification features.

### Methodology

Corpus-based translation researchers have made use of different constructs and measures to study simplification features in translation. One of the reasons hampering previous research on simplification is the use of measures which are tied with individual linguistic features. The use of such measures cannot avoid the issue of “cherry picking”, i.e., intentionally selecting measures to support or reject the hypothesis based on the researcher’s standpoint. There is clearly a need for adopting holistic features to study translational simplification to answer the question of whether and in what specific areas translation tends to be simpler (or possibly more complex) than native language. In order to get a fuller picture of simplification vs complexity in translated texts, the present study adopted five subconstructs of syntactic complexity (length of production units, amount of coordination, amounts of subordination, degree of phrasal sophistication, and overall sentence complexity) from the L2 syntactic complexity analyzer (L2SCA) to study the syntactic complexity of translated and non-translated texts between two corpora (see Table 2 for a summary of these measures and their descriptive statistics). Lu [46] included mean sentence length as a measure of syntactic complexity; however, we excluded this measure as we included mean sentence length as a co-variate to control for possible differences in syntactic complexity between translated and non-translated texts that might be attributed to sentence length.

**Table 2:** Descriptive statistics for the 13 measures in the five subconstructs of syntactic complexity.

Measure and index	COCE				FLOB			
	Min	Max	Mean	SD	Min	Max	Mean	SD
***Length of production unit***								
words per T-unit	8.9	67.5	18.5	6.3	7.6	52.3	19.6	5.6
words per clause	6.6	34.3	12.2	4.0	5.8	22.3	11.8	2.7
***Amount of subordination***								
clauses per T-unit	0.4	3.1	1.7	0.3	0.7	3.5	1.9	0.4
complex T-units per T-unit	1.0	3.4	2.1	0.3	1.3	4.5	2.2	0.4
dependent clauses per clause	0.6	2.5	1.5	0.2	0.9	2.8	1.7	0.3
dependent clauses per T-unit	0.0	0.5	0.3	0.1	0.1	0.6	0.4	0.1
***Amount of coordination***								
coordinate phrases per clause	0.0	1.1	0.5	0.2	0.1	1.7	0.6	0.3
coordinate phrases per T-unit	0.6	1.8	1.1	0.1	0.7	1.6	1.1	0.1
T-units per sentence	0.0	0.7	0.4	0.1	0.1	0.8	0.4	0.1
***Phrasal complexity***								
complex nominals per clause	0.1	2.3	0.5	0.3	0.0	1.9	0.5	0.2
complex nominals per T-unit	0.1	1.6	0.3	0.2	0.0	1.1	0.3	0.1
verb phrases per T-unit	0.5	6.6	2.1	1.0	0.4	6.6	2.4	1.0
***Overall sentence complexity***								
clauses per sentence	0.4	5.0	1.4	0.6	0.3	3.2	1.5	0.5

Results were then converted into MS Excel file for further statistical analysis using R. For statistical analysis, ANOVAs were conducted to examine whether there were significant differences in syntactic complexity (five subconstructs) by translation status (translation vs. non-translation) and genres (press, prose, academic writing and fiction). Pairwise Mann-Whitney tests were computed to measure the differences between each translated and non-translated genre (press, prose, academic writing and fiction) using the 13 measures to obtain a fuller picture of the simplification features.

## Results

Table 2 presents the descriptive statistics of all the 13 measures (in five subconstructs) for the two corpora. As the 13 measures differed vastly in the raw scores, we first turned them (across the two corpora) into z-scores so that they were on the same scale. We first conducted a main analysis to compare the general syntactic complexity, using the z-scores in all the 13 measures as the dependent variable, and corpus (COCE vs. FLOB), genre (news, prose, academic, and fiction) and measures of syntactic complexity (five subconstructs) as interacting independent variables; we also added values of mean sentence length (transformed into z-scores) as a co-variate to control for the possible effect of sentence length on syntactic complexity. We observed a highly significant main effect of corpus, with higher syntactic complexity in the non-translated texts (FLOB) than the translated texts (COCE). There is also a significant difference among the genres, with higher syntactic complexity in academic writing than in fiction in general (see Table 3 and separate analyses below). Measure did not have a significant effect (as we used z-scores where the mean of each measure was 0). There was also a significant interaction between corpus and genre, between corpus and measure, between genre and measure, and among corpus, genre and measure. To further explore these interactions, we next conducted separate analyses comparing the two corpora for each genre and separate analyses comparing the two corpora for each subconstruct of syntactic complexity.

**Table 3:** ANOVA results in the main analysis.

Effect	*F*	*df*	*P*
Corpus	355.9	1	< .001
Genre	1115.6	3	< .001
Measure	0	4	1
Sentence length	5630.9	1	< .001
Corpus:Genre	13.4	3	< .001
Corpus:Measure	88.5	4	< .001
Genre:Measure	61.39	12	< .001
Corpus:Genre:Measure	23.2	12	< .001

Notes: the *df* for the error term was 12959.

**Fig 1:**
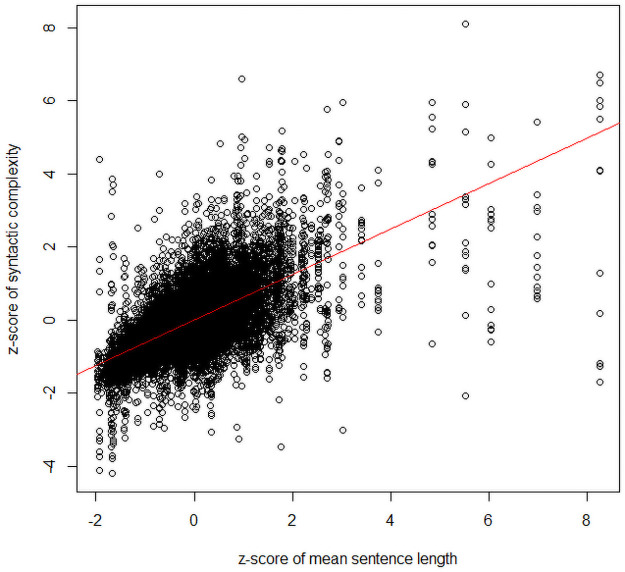
Syntactic complexity (z-score) as a function of number of mean sentence length (z-score) in text.

**Fig 2:**
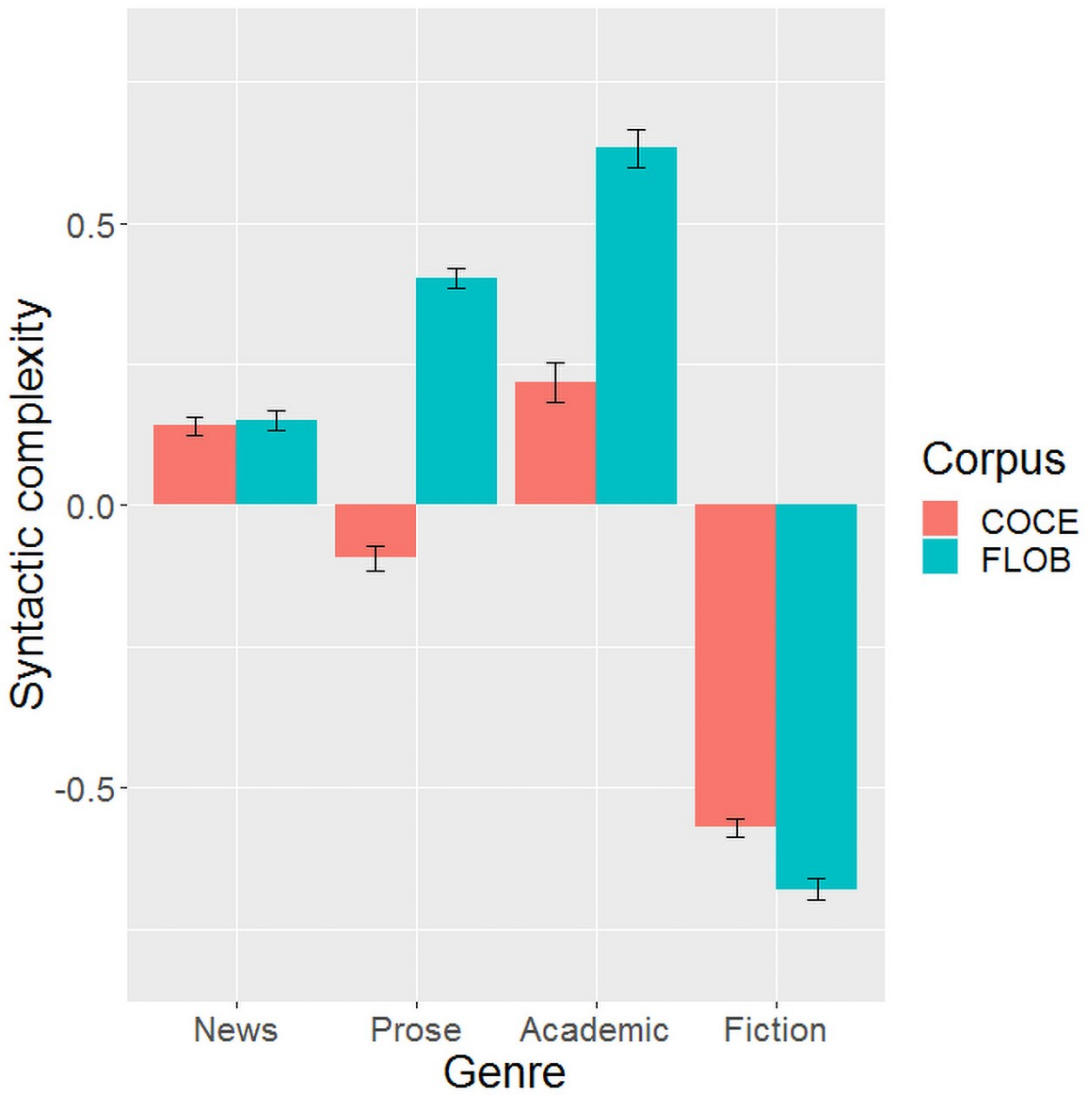
Comparison of the two corpora in syntactic complexity for each genre type. Error bar represents ±SE.

There were four genres in our data: news, prose, academic writing, and fiction. For simplicity, we only included corpus as the independent variable of interest, together with mean sentence length as a co-variate (see Table 4 for the results of the separate ANOVAs). Mean sentence length always produced a significant effect on syntactic complexity, with higher syntactic complexity for longer sentence lengths (see Fig 1). As shown in Fig 2, the FLOB corpus was syntactically more complex than the COCE corpus for prose, academic writing, while COCE is more complex in fiction than FLOB. No significant difference was observed in the genre of news between the two corpora.

**Table 4:** ANOVA results for the by-genre separate analyses.

Effect	News		Prose		Academic		Fiction	
	*F*	*p*	*F*	*p*	*F*	*p*	*F*	*p*
Corpus	0.2	.652	423.3	< .001	89.8	< .001	34.0	< .001
Sentence length	699.3	< .001	2130.5	< .001	471.8	< .001	2249.1	< .001

The *df* was 1 for the effect of corpus and 1 for the effect of sentence length in all analyses. The *df* for the error term was 2285 in news, 5353 in prose, 2077 in academic, and 3273 in fiction (as a result of unequal number of texts in the data).

**Fig 3:**
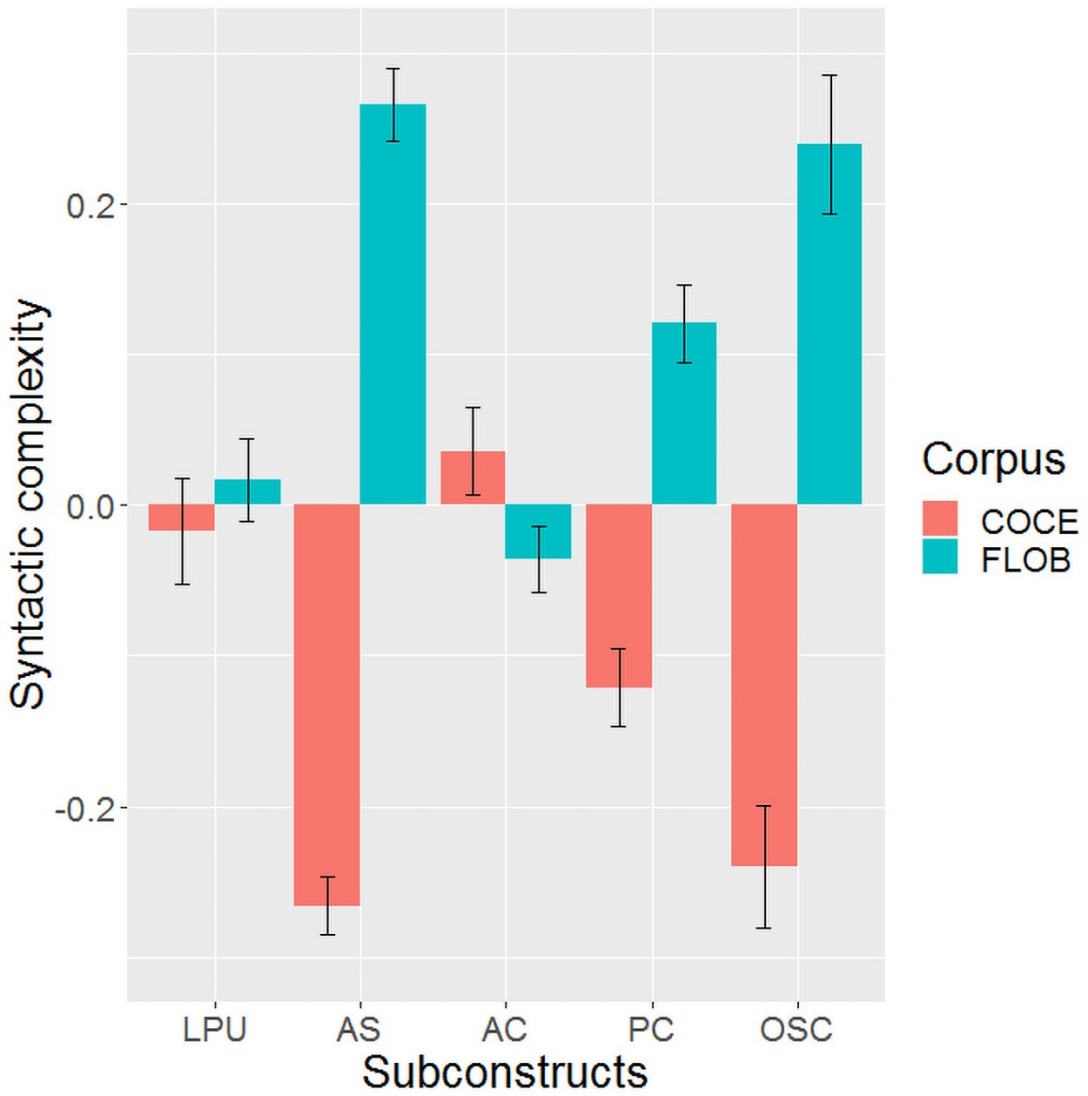
Comparison of the two corpora in syntactic complexity for each measure. Error bar represents ±SE. LPU = Length of Production Unit, AS = Amount of Subordination, AC = Amount of Coordination, PC = Phrasal Complexity, OSC = Overall Sentence Complexity.

We next carried out separate analyses for each subconstruct of syntactic complexity, again using corpus as the independent variable of interest and mean sentence length as a co-variate. Table 5 shows the results of the ANOVAs and Fig 3 shows the comparison of the two corpora for each subconstruct. As can be seen, when sentence length is controlled as a covariate, the length of production unit shows no significant difference between COCE and FLOB. However, sentence length always produced a significant effect in all five subconstructs. The FLOB corpus was syntactically more complex in all subconstructs except in the amount of coordination, where the reverse was observed.

**Table 5:** ANOVA results for the by-subconstruct separate analyses.

Effect	Length of production unit		Amount of subordination		Amount of coordination		Phrasal complexity		Overall sentence complexity	
	*F*	*p*	*F*	*p*	*F*	*p*	*F*	*p*	*F*	*p*
Corpus	1.7	.197	432.3	< .001	5.3	.002	95.3	< .001	86.5	< .001
Sentence length	3960.5	< .001	1667.0	< .001	1075.0	< .001	3420.2	< .001	422.0	< .001

The *df* was 1 for the effect of corpus and 1 for the effect of sentence length in all analyses. The *df* for the error term was 1997 in the analysis of length of production unit, 3997 in the analysis of amount of subordination, 2997 in the analysis of amount of coordination, and 2997 in the analysis of phrasal complexity, 997 in the analysis of overall sentence complexity (as a result of unequal number of texts in the data).

In the following, we compared translated with non-translated text for each genre using each of the 13 complexity measures. As we compared within each measure, we reverted back to their raw scores (instead of using z-scores as we did when we included different measures) and we used Mann-Whitney tests as a result of the data mostly being non-normal.

Table 6 shows the syntactic complexity values (mean ranks) and the results, taking the news genre of the two corpora into account. The overall sentence complexity measured by clauses per sentence shows that translated and non-translated news are not significantly different from each other. Based on the 13 measures of syntactic complexity (see Table 6), the news category shows a mixed result. Of the five significantly different measures, translated news is lower in two measures (dependent clauses per clause, T-units per sentence) but higher in three (clauses per T-unit, coordinate phrases per T-unit, verb phrases per T-unit) than non-translated news. This mixed results also confirmed the ANOVA test that there is no significant difference in overall complexity between translated and non-translated news. Specifically, translated and non-translated news are very similar in the length of production unit which comprises mean length of T-unit and clauses. In terms of subordination and coordination which present mixed results, translated news are complex in certain features but lower in others. In the measures for subordination, while translated news is more complex in clauses per T-unit, non-translated news is more complex in dependent clauses per clause. We can see from Example 1 that both translated and non-translated news contain three clauses, with the non-translated news having 22 words and the translated one having 27 words. In other words, translated news is not less complex than non-translated news in terms of the length of production units. It was found that such a sentence pattern (i.e. higher ratio of clauses per T-unit) is quite common in COCE news. Example 2 shows a typical example from the FLOB corpus in which the dependent clause is much more complex than the one in COCE. This explains why the number of complex dependent clauses (measured by dependent clauses per clause) in the native news component is higher than the translated one. As far as coordination is concerned, translated news is more complex in coordinate phrases per T-unit and less complex in T-unit per sentence. The results here are again also mixed.

**Table 6:** Mann-Whitney tests on syntactic complexity between FLOB news and COCE news.

Measure	FLOB vs. COCE (N = 176)					
	Mean rank		U	z	P	r
***Length of production unit***	FLOB	COCE				
words per T-unit	84.47	92.53	3517	-1.05	.294	.08
words per clause	91.35	85.65	3621.5	-0.74	.459	.06
***Amount of subordination***						
clauses per T-unit	77.49	99.51	2903	-2.87	.004	.22
complex T-units per T-unit	94.6	82.4	3335.5	-1.59	.112	.12
dependent clauses per clause	103.64	73.36	2540	-3.94	< .000	.30
dependent clauses per T-unit	94.54	82.46	3340.5	-1.57	.116	.12
***Amount of coordination***						
coordinate phrases per clause	81.75	95.25	3278	-1.76	.079	.13
coordinate phrases per T-unit	79.38	97.63	3069	-2.37	.018	.18
T-units per sentence	109.06	67.94	2062.5	-5.35	< .000	.40
***Phrasal complexity***						
complex nominals per clause	91.55	85.45	3604	-0.79	.428	.06
complex nominals per T-unit	87.15	89.85	3753.5	-0.35	.726	.03
verb phrases per T-unit	75.85	101.15	2759	-3.29	.001	.25
***Overall sentence complexity***						
clauses per sentence	92.34	84.66	3534.5	-1.00	.318	.08

**Example 1:** Example from news in FLOB and COCE.

FLOB	A02 EDUCATION Secretary Kenneth Clarke confirmed yesterday that he would have resigned from the Government if Mrs Thatcher had carried on in power.
COCE	A01 Chinese Ambassador to the United Kingdom Liu Xiaoming on Sunday said that banning Huawei means back-pedalling for Britain, which would leave the country trailing behind on technology.

**Example 2:** Example from news in FLOB and COCE.

FLOB	B17 Despite the difficulties that surround the treaties which it is hoped will be signed at the Maastricht summit next month, the mood music at Gleneagles implied that monetary union and a single currency were inevitable and even desirable.
COCE	C03 Since China officially started the spokesperson system in 1983, 15 spokespersons of the CPPCC National Committee and eight spokespersons of NPC have spoken to the press, according to China Today.

Table 7 presents the comparison in syntactic complexity between translated and non-translate general prose. As can be seen, the translated genre is lower in all 13 measures and such differences are statistically significant. As demonstrated by the mean ranks, the differences in syntactic complexity in general prose are quite obvious. To a certain extent, the simplification hypothesis is best confirmed in this genre. We give two examples (Examples 3 and 4) with the connective “if” and “despite” to demonstrate the use of the syntactic differences between COCE and FLOB. As can be seen from both examples, FLOB seems to favor long dependent clauses in expressing the idea in general prose, while such elements are relatively shorter in the translation genre.

**Table 7:** Mann-Whitney tests on syntactic complexity between FLOB prose and COCE prose.

Measure	FLOB vs. COCE (N = 352)					
	Mean rank		*u*	*z*	*p*	*r*
***Length of production unit***	**FLOB**	**COCE**				
words per T-unit	229.91	123.09	6087.5	-9.85	< .000	.53
words per clause	205.64	147.36	10359	-5.37	< .000	.29
***Amount of subordination***						
clauses per T-unit	217.10	135.90	8342.5	-7.49	< .000	.40
complex T-units per T-unit	222.37	130.63	7415	-8.46	< .000	.45
dependent clauses per clause	218.78	134.22	8046	-7.80	< .000	.42
dependent clauses per T-unit	220.61	132.39	7725.5	-8.13	< .000	.43
***Amount of coordination***						
coordinate phrases per clause	191.40	161.60	12866	-2.75	.006	.15
coordinate phrases per T-unit	205.97	147.03	10301.5	-5.43	< .000	.29
T-units per sentence	193.26	159.74	12538	-3.09	.002	.17
***Phrasal complexity***						
complex nominals per clause	214.13	138.87	8864.5	-6.94	< .000	.37
complex nominals per T-unit	232.63	120.38	5610	-10.35	< .000	.55
verb phrases per T-unit	214.86	138.14	8737.5	-7.07	< .000	.38
***Overall sentence complexity***						
clauses per sentence	214.73	138.27	8759	-7.05	< .000	.38

**Example 3:** Examples with “if” retrieved from COCE and FLOB general prose.

FLOB	F24 If this is an appropriate description, then it must be assumed that the war had special characteristics which distinguish it from other wars; and indeed the Second World War does form a very important episode in the history of the working classes.
COCE	G61 And if there are limitations, how are they to be determined?

**Example 4:** Examples with “despite” retrieved from COCE and FLOB general prose.

FLOB	F08 Despite the code’s unequivocal assertion that an individual’s capacity to give consent can vary over time, the Appeal Court clearly took a different view.
COCE	G75 But the fields of rice and vegetables did not lie uncultivated despite the men’s contempt for them.

Table 8 presents comparison in syntactic complexity for academic writing between COCE and FLOB. The Mann-Whitney test shows that 11 out of the 13 measures are significantly different between the two corpora. The translated component is higher in four measures (words per clause, coordinate phrases per clause, coordinate phrases per T-unit, complex nominals per clause), but lower in clauses per T-unit, complex T-units per T-unit, dependent clauses per clause, dependent clauses per T-unit, T-units per sentence, verb phrases per T-unit, and clauses per sentence. The overall sentence complexity measured using clauses per sentence also shows that translation is less complex than non-translation in academic writing. The results show that translated academic writing uses less subordination, as reflected by all the four subordination measures. In terms of coordination, the translated academic writing uses more coordinate phrases per clause and per T-units while presents less coordination at the sentence level measured by T-units per sentence. Previous study has identified that ELF academic writing uses more coordination in all three measures than native one [51]. The current study found that translation shares some similarities with ELF academic writing as both language outputs use more coordinate phrases. Example 5 shows the use of coordinate phrases in sentences extracted from FLOB and COCE. As can be seen from Example 5, the translated sentence consists of three coordinate phrases in one sentence, showing that the use of coordinate phrases is quite common in translated texts.

**Table 8:** Mann-Whitney tests on syntactic complexity between FLOB academic writing and COCE academic writing.

Measure	FLOB vs. COCE (N = 220)					
	Mean rank		*u*	*z*	*p*	*r*
***Length of production unit***	FLOB	COCE				
words per T-unit	115.18	105.82	5535	-1.09	.275	.07
words per clause	89.95	131.05	3789	-4.79	< .001	.32
***Amount of subordination***						
clauses per T-unit	135.92	85.08	3253.5	-5.92	< .001	.40
complex T-units per T-unit	137.60	83.40	3069	-6.32	< .001	.43
dependent clauses per clause	135.12	85.88	3341.5	-5.74	< .001	.39
dependent clauses per T-unit	135.71	85.29	3276.5	-5.88	< .001	.40
***Amount of coordination***						
coordinate phrases per clause	79.91	141.09	2685	-7.13	< .001	.48
coordinate phrases per T-unit	86.62	134.38	3423.5	-5.57	< .001	.38
T-units per sentence	121.90	99.10	4795.5	-2.66	.008	.18
***Phrasal complexity***						
complex nominals per clause	97.28	123.72	4595.5	-3.08	.002	.21
complex nominals per T-unit	117.86	103.14	5240	-1.72	.086	.12
verb phrases per T-unit	130.03	90.97	3902	-4.55	< .001	.31
***Overall sentence complexity***						
clauses per sentence	136.43	84.57	3198	-6.04	< .001	.41

**Example 5:** Example retrieved from the academic prose of FLOB and COCE.

FLOB	J72 The levels of lighting will affect the visibility of the instrumentation and equipment and if inadequate may lead to hazardous situations.
COCE	J31 As some nationalities have many branches and sub-branches and their costumes and ornaments are so different, we can only introduce to you the most representative ones or those of higher aesthetic values.

Table 9 indicates that seven out of the thirteen measures are significantly different between FLOB and COCE in this genre. Specifically, translated fiction is more complex in length of production unit measured by mean length of T-unit and clause. As far as subordination is concerned, both translated and non-translated fiction show no significant differences. In other words, translated fiction uses a similar amount of subordination as native fiction. The major differences between the two types of texts lies in coordination measured by coordinate phrases per clause and per T-unit and also the number of T-units per sentence. In all three measures, translated fiction is higher as compared to the non-translated one. As to the overall sentence complexity measured by clauses per sentence, translation is slightly more complex than non-translation but such a difference is not significantly different. The results affirm the ANOVA test that translated fiction is relatively more complex than non-translated fiction as a whole. We chose an example (Example 6) beginning with “it was” in both FLOB and COCE as this phrase is very common in fictional description. We can see that the translated fiction is longer in terms of T-unit and clause. Example 7 shows the use of coordinate phrases used in sentences extracted from the fiction genre of FLOB and COCE.

**Table 9:** Mann-Whitney tests on syntactic complexity between FLOB fiction and COCE fiction.

Measure	FLOB vs. COCE (N = 212)					
	Mean rank		*u*	*z*	*p*	*r*
***Length of production unit***	FLOB	COCE				
words per T-unit	108.44	144.56	5662	-3.93	< .001	.27
words per clause	102.79	150.21	4951	-5.16	< .001	.36
***Amount of subordination***						
clauses per T-unit	123.10	129.90	7510	-0.74	.459	.05
complex T-units per T-unit	120.65	132.35	7201.5	-1.27	.203	.09
dependent clauses per clause	126.05	126.95	7881	-0.1	.922	.01
dependent clauses per T-unit	124.36	128.64	7668	-0.47	.641	.03
***Amount of coordination***						
coordinate phrases per clause	107.68	145.32	5566.5	-4.10	< .001	.28
coordinate phrases per T-unit	109.05	143.95	5739	-3.80	< .001	.26
T-units per sentence	112.13	140.87	6128	-3.13	.002	.22
***Phrasal complexity***						
complex nominals per clause	110.15	142.85	5878	-3.56	< .001	.25
complex nominals per T-unit	112.04	140.96	6115.5	-3.15	.002	.22
verb phrases per T-unit	118.39	134.61	6916	-1.77	.077	.12
***Overall sentence complexity***						
clauses per sentence	118.06	134.94	6874.5	-1.84	.066	.13

**Example 6:** Example of sentence length in fiction between FLOB and COCE.

FLOB	L16 It was less than a year since he had arrived in Port Torquil, saying he wanted to put down roots.
COCE	L01 It was called a fort rather than a village because there had once been a lot of bandits in these parts and, to prevent trouble, it had been placed in splendid isolation on a rise above the river.

**Example 7:** Coordinate phrases in fiction between FLOB and COCE.

FLOB	P14 Marie stiffened and stepped carefully back, distancing herself from the small group.
COCE	P16 What has happened is that gradually, over the years, I have grown strangely like my aunt, and have even assimilated her reticence, her pale face and hands, and her slow way of walking.

## Discussion

In this paper, we studied the syntactic complexity between translated and non-translated texts, with the aim to probe into the simplification features in translation. For this purpose, we compared the English monolingual part of COCE, a Chinese-English corpus, with its non-translation counterpart FLOB using the 13 syntactic complexity measures (five subconstructs) generated by the L2SCA software [46]. This type of research falls into what Chesterman [59] refers to as the T-universals, which characterize the differences between translated texts and non-translated texts. In our study, the ANOVA tests have affirmed that there is a main effect of translation status, suggesting native texts are more complex than translated texts; and there is also an interaction, suggesting that the corpus effect differs between genres. Results show that translated texts in general are relatively lower in syntactic complexity as evidenced by four of the five complexity subconstructs. On the other hand, when the four genres are compared against each other between the two corpora, we found that the complexity level changes and present a different pattern than that of the whole corpus, suggesting that genre is an important variable in affecting the complexity level of the two types of texts. Pairwise comparisons of news, general prose, academic writing, and fiction using Mann-Whitney tests also show that these four genres are not consistent in terms of the specific measures of syntactic complexity. Specifically, translated and native news are quite homogenous and there is no significant difference between them when all measures were treated as a whole. Translated news is more complex in certain measures and simpler in others than non-translated news. On the other hand, translated general prose, and translated academic writing seem to follow the simplification trend with most complexity measures lower than their native counterparts. In fiction, the results suggest that translated fiction, which uses more coordination and similar amount of subordination, is more complex than the non-translated one. These differences present a clear picture that English translations from Chinese is unique, genre-specific and possibly governed by some source language norms. Our research findings echoed previous studies which identified that the simplification level of translated texts is not universal and to a large extent subject to the source language influence [55, 60].

As can be seen from the current study, simplification has many levels and comprises a variety of linguistic features. Although the overall comparison between translation and non-translation as a whole has provided support for the simplification hypothesis, further comparisons of the four subgenres revealed mixed results. For example, the news genre shows similarities in syntactic complexity in both translated and non-translated corpora. As a non-literary genre, news writing emphasizes practicality and instantaneity and follows a rather rigid writing format, i.e. the inverted-pyramid structure. To a certain extent, such a genre would transcend the writing traditions of the source language socio-cultural norms. The ANOVA test has shown that genre plays an equally essential role as translation status in affecting the complexity level of texts. For many years, the translation field has prioritized the translation status in corpus-based investigations at the expense of other important variables such as genres. Based on the findings of the current research, we contend that genre should be treated as an important variable in the quest for simplification as well as other TUs candidates.

Translation is both a cognitive endeavor carried out inside the mind of the translator and a social conduct cutting across languages and cultures [15]. One widely accepted explanation has been the Hypothesis of Gravitational Pull [33, 34] which draws on both bilingual theory and cognitive linguistics. The hypothesis states that target-language prototypical or highly salient linguistic forms would exert a pull on a translator’s decision-making processes, also known as magnetism effect. The prototypical language features that are stored in the translator’s mind can lead to simplification in the translation. Conversely, the source text would also exert a counter-pull resisting the target-text force and lead to interference (the gravitational pull effect). In addition to this effect is the connectivity effect which will take place due to the impact of high frequency co-occurrence of translation equivalents in the source and target languages. The interrelation and interplay of the three forces will result in the make-up of the translated language. Although this model was mainly used to explain translation universals which involve linguistic features at the lexical level (e.g. unique items hypothesis which claims translations tend to contain fewer “unique items” than comparable non-translated texts [61, 62], it is believed that this model is also applicable to syntactic features based on the findings of the current study. Specifically, we can see that the news genre is different from the other three genres and shows more homogeneity with non-translated news. We contend that the Chinese source text norms governing news writing are similar to English news writing norms. In this way, the English translated news resembles the non-translated one even undergoing the translation process, which shows that the “connectivity effect” is at work. On the other hand, the norms of other text types between English and Chinese might be vastly different. In this case, the gravitational pull effect prevails over the other two effects in affecting the profiling of the translated language. Take fiction as an example, it was found that FLOB fiction contains many more dialogues than COCE, while COCE contains relatively more descriptive language. This probably explains the reason why translated fiction is comparatively more complex than non-translated fiction.

Previously, most research on TUs seems to put a focus on the study of literary texts while ignoring the non-literary texts. This is also the case many studies using TEC (Translational English Corpus) held at Manchester University which has been criticized as skewed towards the literary genre. Based on our research findings, we found that the quest for translation universals cannot be totally free from the source language or genre interference. Taking genre into account, the current research reveals some interesting findings that might otherwise remain hidden in corpus-based studies where genre is not taken into account. In a sense, the complexity level of texts is a combined effect of cognitive factors and translator’s decision-making process [63]. Our research findings have affirmed the proposal by Kruger and Van Rooy [45] that the concept of ‘translated language’ needs to be addressed together with genre or register in order to get more nuanced interpretations of the features of translated language.

## Conclusion

This study was aimed at identifying the simplification features in translated texts using syntactic complexity measures to compare between translated English from Chinese and the non-translated native English writing. By systematically studying syntactic complexity in four major genres between the translated and non-translated texts, our study has lent some support for the simplification hypothesis; however, it also identifies that genre is an important variable contributing to the different profiling between translated and non-translated texts apart from translation status. Our study also shows the potential of using syntactic complexity measures for corpus-based investigations of simplification and possibly other TUs. However, the findings from the current research are only limited to Chinese-English translation. Previous research [64] has shown variation in syntactic complexity among texts produced by writers with different L1s. As COCE contains translated texts mostly done by Chinese-speaking translators, the findings concerning the different subconstructs of syntactic complexity are strongly related to such a variable. Future studies can be conducted with other language pairs to provide better insight into such an issue.
